# *Plasmodium falciparum* parasitaemia in the first half of pregnancy, uterine and umbilical artery blood flow, and foetal growth: a longitudinal Doppler ultrasound study

**DOI:** 10.1186/1475-2875-11-319

**Published:** 2012-09-10

**Authors:** Jennifer B Griffin, Victor Lokomba, Sarah H Landis, John M Thorp, Amy H Herring, Antoinette K Tshefu, Stephen J Rogerson, Steven R Meshnick

**Affiliations:** 1 Department of Epidemiology, Gillings School of Global Public Health, University of North Carolina, McGavran-Greenberg Hall, Campus Box 7435, Chapel Hill, 27599-7435, USA; 2 Ecole de Santé Publique, Faculté de Medecine, Université de Kinshasa, Kinshasa, Republique Démocratique du Congo; 3 Oncology Research and Development, GlaxoSmithKline, Middlesex, TW8 9GS, UK; 4 Department of Obstetrics and Gynecology, University of North Carolina, Chapel Hill, 27599, USA; 5 Department of Biostatistics, Gillings School of Global Public Health, University of North Carolina, Chapel Hill, 27599, USA; 6 Department of Medicine (RMH/WH), The University of Melbourne, Royal Melbourne Hospital, Parkville, 3050, Australia

**Keywords:** Doppler, Malaria parasitaemia, Placenta, Pregnancy, Umbilical artery, Uterine artery, Foetal growth

## Abstract

**Background:**

During early pregnancy, the placenta develops to meet the metabolic demands of the foetus. The objective of this analysis was to examine the effect of malaria parasitaemia prior to 20 weeks’ gestation on subsequent changes in uterine and umbilical artery blood flow and intrauterine growth restriction.

**Methods:**

Data were analysed from 548 antenatal visits after 20 weeks’ gestation of 128 women, which included foetal biometric measures and interrogation of uterine and umbilical artery blood flow. Linear mixed effect models estimated the effect of early pregnancy malaria parasitaemia on uterine and umbilical artery resistance indices. Log-binomial models with generalized estimating equations estimated the effect of early pregnancy malaria parasitaemia on the risk of intrauterine growth restriction.

**Results:**

There were differential effects of early pregnancy malaria parasitaemia on uterine artery resistance by nutritional status, with decreased uterine artery resistance among nourished women with early pregnancy malaria and increased uterine artery resistance among undernourished women with early pregnancy malaria. Among primigravidae, early pregnancy malaria parasitaemia decreased umbilical artery resistance in the late third trimester, likely reflecting adaptive villous angiogenesis. In fully adjusted models, primigravidae with early pregnancy malaria parasitaemia had 3.6 times the risk of subsequent intrauterine growth restriction (95% CI: 2.1, 6.2) compared to the referent group of multigravidae with no early pregnancy malaria parasitaemia.

**Conclusions:**

Early pregnancy malaria parasitaemia affects uterine and umbilical artery blood flow, possibly due to alterations in placentation and angiogenesis, respectively. Among primigravidae, early pregnancy malaria parasitaemia increases the risk of **intrauterine** growth restriction. The findings support the initiation of malaria parasitaemia prevention and control efforts earlier in pregnancy.

## Background

In malaria endemic areas, the World Health Organization recommends prevention and control strategies for malaria parasitaemia in pregnancy, including case management of malaria parasitaemia and anaemia; insecticide-treated nets (ITNs); and, at least two doses of intermittent preventive treatment in pregnancy (IPTp) with sulphadoxine-pyrimethamine after the awareness of foetal movement [1] (from approximately 17–19 weeks’ gestation [2]).The peak prevalence of malaria parasitaemia in pregnancy occurs from 13 to 20 weeks’ gestation [3], mostly prior to the first dose of IPTp [1].

During this critical period of early pregnancy, the placenta develops to meet the growing metabolic demands of the foetus. Extravillous trophoblast cells invade and remodel the uterine spiral arteries, increasing uterine artery blood flow from the maternal circulation to the maternal side of the placenta. Concurrently, villous angiogenesis leads to increased umbilical artery blood flow from the foetus to the foetal side of the placenta.

Doppler ultrasound allows the non-invasive investigation of utero- and foeto-placental blood flow and resistance. The assessment of uterine artery blood flow reflects the extent of trophoblast invasion of the spiral arteries [4,5]. Abnormal uterine artery resistance is associated with pre-eclampsia, intrauterine growth restriction (IUGR) and adverse pregnancy outcomes [6], while increased umbilical artery resistance is associated with foetal distress and IUGR [7,8]. Ultrasound can assess foetal biometry measurements, which can be used to identify IUGR foetuses by comparing estimated foetal weights to established foetal growth standards.

It has been previously hypothesized that malaria parasitaemia in early pregnancy disrupts trophoblast invasion [9], leading to diminished utero-placental blood flow and, ultimately, intrauterine growth restriction. The objective of this analysis was to examine the hypothesis that early pregnancy malaria parasitaemia leads to subsequent changes in uterine artery and umbilical artery resistance indices, indicating alterations in placentation and villous angiogenesis, respectively. The effect of early pregnancy malaria parasitaemia on the risk of subsequent IUGR was also examined. The effect of early pregnancy malaria parasitaemia on uterine artery resistance was modified by maternal nutritional status, with increased resistance among undernourished women with early malaria infection and decreased resistance among nourished women with early malaria infection. Among primigravidae, early pregnancy malaria parasitaemia decreased umbilical artery resistance in the late third trimester. After controlling for confounders, primigravidae with early pregnancy malaria parasitaemia had nearly four times the risk of subsequent intrauterine growth restriction compared to the referent group of multigravidae with no early pregnancy malaria parasitaemia.

## Methods

### Study population

The longitudinal cohort consisted of pregnant women presenting for first antenatal care at Binza Maternity Hospital in Kinshasa, Democratic Republic of Congo between May 2005 and May 2006, as previously described [10,11]. In brief, 182 pregnant women ≥18 years, with non-hypertensive, non-anomalous singleton pregnancies; and, ultrasound-derived gestational age of ≤22 weeks were enrolled and followed until delivery. Enrolled women were provided with ITNs and received IPTp with sulphadoxine-pyrimethamine twice during pregnancy (between 16–27 and 28–32 weeks’ gestation) regardless of malaria parasitaemia status. All women with microscopy positive malaria parasitaemia were treated throughout follow-up.

All participants provided written informed consent to participate in the study. This study was approved by the Institutional Review Boards of the University of North Carolina at Chapel Hill and the Kinshasa School of Public Health.

For the current analysis of early pregnancy malaria parasitaemia exposure, five HIV-positive participants and 49 participants with no malaria parasitaemia exposure data ≤20 weeks’ gestation were excluded, leaving an analytic sample of 548 antenatal visits after 20 weeks’ gestation from 128 women, which included foetal biometric measures and interrogation of uterine and umbilical blood flow. Excluded participants did not significantly differ from the analytic population in age, gravidity, foetal sex, or socio-economic status (SES), but were more likely to have a low level of education and to be undernourished (data not shown). There were 6 probable recrudescent malaria episodes which occurred within 14 days of a previous infection, despite receiving treatment. Recrudescent malaria episodes were excluded from all analyses. There was no association between being a recrudescent case and parasite density as reflected by qPCR Ct-value (data not shown). Participants in the analytical sample were enrolled at a median of 18 weeks’ gestation (interquartile range [IQR]: 16, 19) and were followed up for a median of 19 weeks (IQR: 17, 21). Study participants had a median of seven follow-up visits (IQR: 6, 8), with five ultrasound scans (IQR: 4, 5) from enrolment to delivery.

### Clinical, laboratory, and ultrasound procedures

Baseline data were collected in an interview (socio-demographics, medical history); medical examination (maternal anthropometrics, blood pressure, pulse, temperature); ultrasound examination (estimation of gestational age and foetal weight); and laboratory testing (malaria parasitaemia thick and thin smears; and, filter-paper dried blood spot samples of peripheral blood) as previously described [11]. At monthly antenatal care visits, women repeated ultrasound, medical, and laboratory examinations (including malaria parasitaemia thick and thin smears and filter-paper dried blood spot samples). Quantitative real-time polymerase chain reaction (qPCR) was conducted to detect *Plasmodium* species from dried blood spot samples and *Plasmodium* positive samples were speciated as previously described [12].

Colour pulsed-wave Doppler ultrasound was used to interrogate the flow velocity waveforms in the left and right uterine arteries and the umbilical artery using standard techniques. The external iliac artery and uterine artery located medial to it were identified using colour flow settings. Flow velocity waveforms were obtained from the uterine artery near the iliac vessel prior to division of the uterine artery into branches. For left and right uterine artery analyses, Doppler indices were measured from the Doppler signal, with qualitative assessment of early diastolic notching. For the umbilical artery, the flow velocity waveform was obtained from the free flowing portion of the umbilical cord and Doppler indices and presence of absent or reversed end-diastolic flow were recorded. All ultrasound measurements were taken by a single, trained obstetrician-gynaecologist (VL) on the GE Logiqbook Ultrasound System (GE Medical Systems, Milwaukee, WI, USA).

### Variable definitions

The exposure variable, “early pregnancy malaria parasitaemia”, was a binary, time-independent measure representing whether a woman was ever qPCR positive for peripheral *Plasmodium falciparum* malaria parasitaemia ≤20 weeks’ gestation. Quantitative PCR of peripheral dried blood spots rather than blood microscopy was used due to the higher detection threshold of microscopy and the potential for poor microscopy sensitivity and specificity [13].

Outcome variables for placental blood flow included the continuous, time-dependent mean of the left and right uterine artery resistance index (RI); and, the umbilical artery RI after 20 weeks’ gestation. The RI is defined as (peak systolic velocity – end diastolic velocity)/peak systolic velocity. The resistance index was selected as the outcome for several reasons: RI values are constrained between 0 and 1; demonstrate the least variance of the Doppler indices under identical hemodynamic conditions; are frequently used in clinical settings; and, have a truncated normal distribution, making the RI amenable to parametric statistical analyses [14].

IUGR after 20 weeks’ gestation was also examined. IUGR was a binary, time-dependent outcome. At each visit, women were classified as having an IUGR episode if, at that visit, their foetus was <10^th^ percentile of sonographically estimated foetal weight for gestational age in completed weeks, using Hadlock’s algorithm to estimate foetal weight [15] and the ultrasound-derived, longitudinal, sex-specific Johnsen foetal growth standard [16]. The Johnsen growth standard was utilized because it is a longitudinally-derived, sex-specific nomogram. The Landis growth standard developed in the current study cohort [17] was not utilized due to its self-referential nature. Repeated episodes of IUGR were also examined. “Repeat IUGR” was a binary, time-dependent outcome defined as IUGR (as previously defined), with ≥2 total IUGR episodes during pregnancy.

The continuous time metric, gestational age in weeks, was back-calculated from the first ultrasound using Hadlock’s algorithm [18]. Ultrasound estimated gestational age has been shown to provide the best estimate of gestational age, even when last menstrual period dates are considered certain [19].

Based on previous literature of the association between malaria parasitaemia during pregnancy and foetal growth, the potential modifying effects of primigravidity (*vs.* multigravidity), female foetal sex (*vs.* male), and baseline maternal mid-upper arm circumference (MUAC) ≤24.3 cm (i e, the lowest quartile of the full study population) (*vs.* “normal” MUAC) were examined. MUAC is a proxy for pre-pregnancy weight [20]. Potential confounders included maternal age, education, foetal sex, and socioeconomic status (SES). “Low SES” was defined as a binary composite variable, with unemployed women (or their partners) living in a home with few material assets (no toilet, no water, no electricity) categorized as “low SES”.

### Statistical analysis

For linear mixed effect (LME) models, mean differences were reported and population average growth curves were plotted to describe the unadjusted and adjusted effect of early pregnancy malaria parasitaemia on uterine artery RI and umbilical artery RI. To describe the effect of early pregnancy malaria parasitaemia on IUGR, unadjusted and adjusted risk ratios (RRs) and 95% CIs were modelled using log-binomial regression generalized estimating equation (GEE) regression models with an exchangeable working correlation matrix. LME and GEE regression models are appropriate for longitudinal analyses as they account for the correlation between repeated measures in an individual [21,22].

For multivariable modelling of the effect of early pregnancy malaria parasitaemia on uterine artery RI and umbilical artery RI, a fully adjusted LME model with a random intercept was initially modelled. Linearity between gestational age and the outcome variables was considered by examining polynomial transformations of gestational age using −2 log-likelihood (−2LL) tests for nested models using maximum likelihood estimation (*a priori* cut-off of p < 0.10). The addition of additional random effects was tested using -2LL tests with a mixed chi-square distribution (*a priori* cut-off of p < 0.05). In the final uterine artery RI and umbilical artery RI models random intercepts and slopes were included for each woman. For log-binomial GEE models, a crude model fitted for the early pregnancy malaria parasitaemia exposure variable was utilized. Next, simple log-binomial models with early pregnancy malaria parasitaemia, a theorized effect measure modifier, and their product interaction term were constructed. If the product interaction term was significant using the Wald test (*a priori* cut-off of 0.10), it was considered an effect measure modifier and was included in the multivariable model. For multivariable modelling, a fully adjusted log-binomial model was initially modelled, including the early pregnancy malaria parasitaemia exposure, the significant product interaction terms and potential confounders of the association between malaria parasitaemia and IUGR. A backward elimination approach was used to assess effect measure modification. In LME models, -2LL tests were used to assess the contribution of the product interaction terms to the model using a maximum likelihood approach; in GEE models, Wald tests were used (*a priori* cut-offs of p < 0.10). Potential modifiers that did not modify the association between malaria parasitaemia and the outcome were assessed as potential confounders. Covariates were considered confounders if the change in the co-efficient of the main exposure was greater than 10%, within strata of modifiers. Restricted maximum likelihood was used for final LME model estimates, as is common practice [23]. All analyses were performed using SAS software (SAS, Cary, NC, USA).

## Results

### Baseline characteristics of the study population

Among the analytical sample of 128 pregnant women, 30% were ever qPCR positive for malaria parasitaemia and 21% had early pregnancy malaria parasitaemia (i.e. prior to 20 weeks’ gestation) (Table 1). Primigravidae with early pregnancy malaria parasitaemia had a mean qPCR Ct-value of 24.1 (SD 12.9), while multigravidae with early pregnancy malaria parasitaemia had a mean value of 31.4 (SD 10.7), indicating higher levels of P. falciparum nucleic acid levels in the samples from primigravidae (p = 0.17; data not shown). Similarly, no primigravidae were found to have sub microscopic parasitaemia, while in 30% of multigravida parasitaemia was submicroscopic. These semi-quantitative findings indicate that while the frequency of malaria infection was similar in primigravidae and multigravidae (Table 1), primigravidae had a higher parasite burden compared to multigravidae. Nearly all malaria infections were sub-clinical, with only one infection accompanied by fever.

**Table 1:** Baseline characteristics of pregnant women by early pregnancy malaria parasitaemia status (n = 128). Kinshasa, Democratic Republic of Congo, 2005–2006

**Characteristic**	**Total (n = 128)**	**Early pregnancy malaria parasitaemia (n = 27)**	**No early pregnancy malaria parasitaemia (n = 101)**	***P***^***a***^
	**N (%)**	**N (%)**	**N (%)**	
Maternal age				
≥30	48 (38)	6 (22)	42 (42)	0.13
25-29	40 (31)	9 (33)	31 (31)	
<25	40 (31)	12 (44)	28 (28)	
Gravidity				
Primigravidae	34 (27)	6 (18)	28 (82)	0.56
Multigravidae	94 (73)	21 (22)	73 (78)	
Foetal sex				
Female	70 (55)	17 (24)	53 (76)	0.36
Male	57 (45)	10 (18)	47 (83)	
Low SES				
Yes	109 (85)	23 (21)	86 (79)	0.99
No	19 (15)	4 (21)	15 (79)	
Low education				
Yes	55 (43)	16 (29)	39 (71)	0.054
No	74 (57)	11 (15)	62 (85)	
Baseline MUAC <24.3				
Yes	28 (22)	6 (21)	22 (79)	0.96
No	100 (78)	21 (21)	79 (79)	

SES, socio-economic status; MUAC, mid-upper arm circumference.

a. *P* values from χ^2^ tests.

The mean age of the study population was 27.6 years (SD: 5.1; range 18–42). Of the study participants, 27% of women were primigravidae and 22% of women had a low baseline MUAC. More than half of women (55%) were pregnant with a female foetus. Women with and without early pregnancy malaria parasitaemia had approximately balanced gravidity, foetal sex, SES and low baseline MUAC. There was a non-significant trend towards greater risk of early pregnancy malaria parasitaemia among younger women and women with less education.

### Uteroplacental blood flow and foetal growth

**Figure 1:**
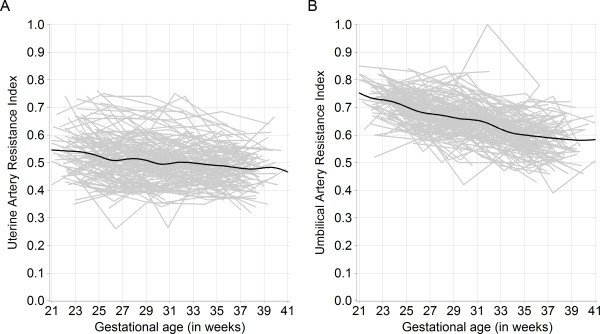
**Time plot of mean uterine and umbilical artery resistance index against gestational age.** Population average smoothed trend line (in black) (n = 128, with 544 visits). Kinshasa, Democratic Republic of Congo, 2005–2006. **A.** Uterine artery resistance index against gestational age in weeks. **B.** Umbilical artery resistance index against gestational age in weeks.

Individual trajectories of the continuous outcomes, uterine artery RI and umbilical artery RI, were plotted in order to examine intra- and inter-individual variability over gestational age in weeks (Figure 1). There is a slightly negative slope for uterine artery RI over gestational age (Figure 1, Panel A). Umbilical artery RI was relatively higher in early pregnancy and decreased more rapidly over the course of pregnancy (Figure 1, Panel B). Of the 128 women, 44 (34%) ever had an IUGR episode after 20 weeks’ gestation, 55% had one episode and 27% had two episodes, with 8 women having three or more episodes. Most IUGR episodes occurred in the late second and early third trimesters.

### Early pregnancy malaria parasitaemia and uterine artery resistance

**Figure 2:**
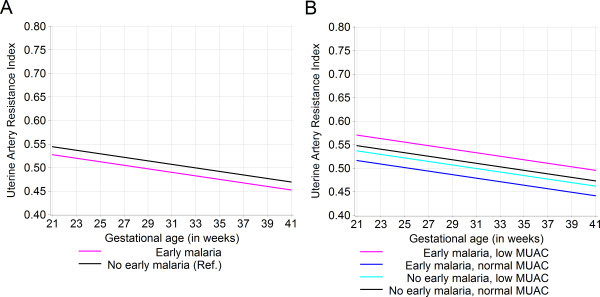
**Population average growth curves for uterine artery resistance index by early pregnancy malaria parasitaemia exposure.** Kinshasa, Democratic Republic of Congo, 2005–2006. **A.** The unadjusted effect of early pregnancy malaria parasitaemia on mean uterine artery resistance index (n = 547 visits). **B.** The adjusted effect of early pregnancy malaria parasitaemia on mean uterine artery resistance index by maternal mid-upper arm circumference (MUAC). Model adjusted for gravidity and foetal sex; product interaction term (early pregnancy malaria parasitaemia * low MUAC) -2LL test p = 0.026, with 1 df (n = 544 visits). The overall standard deviation for uterine artery resistance index was 0.089.

There was no crude effect of early pregnancy malaria parasitaemia on uterine artery RI (mean difference: -0.017; 95% CI: -0.045, 0.011) (Figure 2, Panel A). In the adjusted model, the effect of early pregnancy malaria parasitaemia on uterine artery RI varied by maternal nutritional status (Figure 2, Panel B). Compared to the referent group of women with no early pregnancy malaria parasitaemia with normal MUAC, early pregnancy malaria had a significant independent effect on uterine artery RI of −0.032 (95% CI: -0.062, -0.0006). Low MUAC at baseline, indicating maternal undernutrition, did not have a significant independent effect on uterine artery RI (mean difference: -0.011; 95% CI: -0.042, 0.019). The joint mean difference in uterine artery RI for women with early pregnancy malaria parasitaemia and low MUAC was +0.022 (95% CI: -0.031, 0.076), with a significant difference between undernourished and nourished women with early pregnancy malaria (−2LL test for product interaction term p = 0.026). Thus, early pregnancy malaria parasitaemia led to a lasting significant increase in uterine artery RI of 4% among undernourished women, but caused a decrease in uterine artery RI of 6% among nourished women, compared to the referent group of normally nourished women with no early pregnancy malaria. Foetal sex and gravidity were not found to modify the association between early pregnancy malaria parasitaemia and uterine artery RI.

### Early pregnancy malaria parasitaemia and umbilical artery resistance

**Figure 3:**
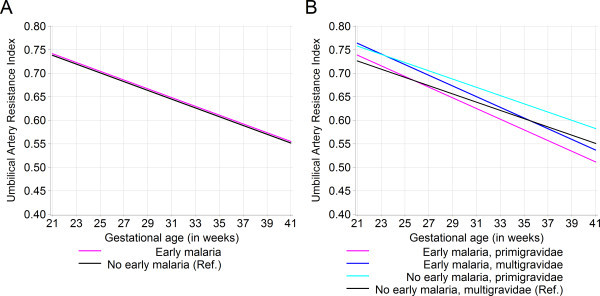
**Population average growth curves for umbilical artery resistance index by early pregnancy malaria parasitaemia exposure.** Kinshasa, Democratic Republic of Congo, 2005–2006. **A.** The unadjusted effect of early pregnancy malaria parasitaemia on umbilical artery resistance index (n = 547 visits). **B.** The adjusted effect of early pregnancy malaria parasitaemia on umbilical artery resistance index by gravidity. Model adjusted for foetal sex and low education; interaction terms: (early pregnancy malaria parasitaemia * gestational age in weeks) -2LL test p = 0.025; (early pregnancy malaria parasitaemia * primigravidae) -2LL test p = 0.025, with 1 df (n = 540 visits). The overall standard deviation for UA RI was 0.089.

There was no crude effect of early pregnancy malaria parasitaemia on umbilical artery RI (Figure 3, Panel A). In the adjusted model, the effect of early pregnancy malaria parasitaemia on umbilical artery RI varied over time and by gravidity (Figure 3, Panel B). Compared to the referent group of multigravidae with no early pregnancy malaria parasitaemia, the mean difference in umbilical artery RI for early pregnancy malaria parasitaemia varied from 0.038 (95% CI: 0.0065, 0.069) at 21 weeks’ gestation to −0.0087 (95% CI: -0.04, 0.03) at 39 weeks’ gestation. The mean difference in umbilical artery RI was 0.032 (95% CI: 0.012, 0.052) for primigravidae. The mean difference in umbilical artery RI for the joint effect of early pregnancy malaria parasitaemia and primigravidity varied from 0.012 (95% CI: -0.030, 0.055) at 21 weeks’ gestation to −0.032 (95% CI: -0.079, 0.011) at 39 weeks’ gestation. Thus, multigravidae with early pregnancy malaria parasitaemia had elevated umbilical artery RI that decreased to levels similar to unexposed multigravidae during the third trimester. Among primigravidae, early pregnancy malaria parasitaemia led to decreased umbilical artery RI during the late third trimester, particularly compared to primigravidae with no early pregnancy malaria parasitaemia. Both multigravidae and primigravidae with malaria infection had a greater negative umbilical artery RI slope than uninfected women.

### Early pregnancy malaria parasitaemia and intrauterine growth restriction

In the unadjusted model, early pregnancy malaria parasitaemia was associated with 1.8 times the risk of subsequent IUGR during pregnancy (95% CI: 1.1, 2.9) (Table 2). In the adjusted model, the effect of early pregnancy malaria parasitaemia was found to vary by gravidity (Wald test for product interaction term: p = 0.035). Primigravidae with early pregnancy malaria parasitaemia had 3.6 times the risk of subsequent IUGR compared to the referent group of multigravidae with no early pregnancy malaria parasitaemia (95% CI: 2.1, 6.2). Early pregnancy malaria parasitaemia was associated with a small increased risk of IUGR among multigravidae, but this finding did not reach statistical significance. In the unadjusted model for repeat IUGR, early pregnancy malaria parasitaemia was associated with 2.2 times the risk of repeat IUGR episodes. In the adjusted model, the risk of repeat IUGR episodes among primigravidae with early pregnancy malaria parasitaemia was 5.6 (95% CI: 2.8, 11.3). The increased risk for repeat IUGR with early pregnancy malaria parasitaemia likely reflects the better ability of repeated episodes of <10^th^ percentile sonographically estimated foetal weight to capture truly IUGR foetuses than a single episode.

**Table 2:** The effect of early pregnancy malaria parasitaemia on subsequent intrauterine growth restriction (IUGR)

	**Early pregnancy malaria parasitaemia**		**No early pregnancy malaria parasitaemia**	
	**RR**	**(95% CI)**	**RR**	**(95% CI)**
IUGR				
Unadjusted	1.8	(1.1, 2.9)	1.0	Ref.
Adjusted^ab^				
Primigravidae	3.6	(2.1, 6.2)	1.1	(0.6, 1.9)
Multigravidae	1.4	(0.8, 2.5)	1.0	Ref.
Repeat IUGR				
Unadjusted	2.2	(1.1, 4.2)	1.0	Ref.
Adjusted^cd^				
Primigravidae	5.6	(2.8, 11.3)	1.2	(0.5, 3.0)
Multigravidae	1.4	(0.6, 3.6)	1.0	Ref.

GEE, generalized estimating equation; IUGR, intrauterine growth restriction; RR, risk ratios; MUAC, mid-upper arm circumference.

a. Adjusted for foetal sex and low baseline MUAC.

b. Interaction term (early pregnancy malaria parasitaemia*primigravidae) p = 0.035.

c. Adjusted for foetal sex and low baseline MUAC.

d. Interaction term (early pregnancy malaria parasitaemia*primigravidae) p = 0.06.

GEE log-binomial model estimated unadjusted and adjusted risk ratios (RR) for the effect of early pregnancy malaria parasitaemia on subsequent IUGR episodes and repeat IUGR episodes (n = 544). Adjusted models stratified by gravidity. Kinshasa, Democratic Republic of Congo, 2005–2006.

## Discussion

This study provides evidence that early pregnancy malaria parasitaemia may alter placentation and lead to intrauterine growth restriction. These observations have important implications for future research directions and could ultimately inform the timing of malaria parasitaemia prevention and control efforts. The effects of early pregnancy malaria parasitaemia could be seen on both uterine and umbilical artery blood flow, indicating alterations in placentation and villous angiogenesis, respectively. Primigravidae with early pregnancy malaria parasitaemia had greater than three times the risk of subsequent IUGR and greater than five times the risk of repeat IUGR episodes compared to multigravidae with no early pregnancy malaria parasitaemia. The effects of early pregnancy malaria parasitaemia on foetal growth were less pronounced among multigravidae, suggesting that the effects of early pregnancy malaria parasitaemia are worse for primigravidae than multigravidae, as described at term [24]. This difference may also reflect the increased intensity of infection among primigravidae compared to multigravidae, as previously suggested [25]. While the findings of the current study, designed as a pilot for a larger subsequent trial, should be interpreted with caution due to small sample size, the results support recent evidence of early pregnancy malaria parasitaemia and restricted foetal growth from Thailand [26].

Differential effects of early pregnancy malaria parasitaemia on uterine artery RI between women with low and normal MUAC were found, suggesting important interactions between malaria parasitaemia and nutritional status on placental development [4,5]. The effects of malaria parasitaemia on foetal growth have previously been shown to vary by nutritional status [11], but the joint effects of malaria parasitaemia and nutritional status during pregnancy on the foetus are not well understood. Extravillous trophoblast invasion is highly regulated and is critical to establishing physiologic uteroplacental blood flow. Malaria parasitaemia in the placenta could dysregulate trophoblast invasion via relative placental hypoxia [27,28]; increases in inflammatory cells and mediators (such as TNFα) [29-31]; functional folate deficiency [32,33]; and/or, increased complement activation [34,35]. Maternal nutritional status at baseline did not have a significant, independent effect on uterine artery resistance in the current study. However, maternal nutritional status has been demonstrated to affect placental development, with differential effects based on the timing and type of nutritional insult [36-38]. Decreased uterine artery resistance (i.e. increased uterine artery blood flow) with early pregnancy malaria or undernutrition may be an adaptive response to increase oxygen and nutrients to the placenta/foetus [39]. However, among undernourished women with early pregnancy malaria, these adaptations may not occur, leading to increased uterine artery resistance.

Increased uterine artery resistance has been previously shown to be associated with restricted foetal growth [5]. In the fully adjusted IUGR model, early pregnancy malaria was found to be associated with IUGR among primigravidae. However, maternal nutritional status at enrolment was not found to be independently associated with IUGR, as previously demonstrated in the current cohort [40]. Neither was there found to be an interaction between early pregnancy malaria and maternal undernutrition at baseline in the IUGR models. These findings indicate that, in this cohort, early pregnancy malaria had a more important impact on foetal growth than maternal nutritional status at baseline.

Altered uterine artery resistance with early pregnancy malaria parasitaemia indicates that placental malaria parasitaemia may be associated with diseases of trophoblast hypo-invasion, such as pre-eclampsia [41]. Evidence consistent with increased trophoblast invasion among nourished women with early pregnancy malaria parasitaemia was also found. Placental bed biopsies are necessary to determine if the effects of early pregnancy malaria parasitaemia on trophoblast invasion are physiological or pathological. The findings demonstrate that malaria parasitaemia prior to 20 weeks’ gestation affects placentation and may contribute to poor pregnancy outcomes due to dysregulation of trophoblast invasion.

Among primigravidae, early pregnancy malaria parasitaemia led to decreased umbilical artery resistance, which may indicate increased angiogenesis in the villous tree of the placenta [42]. Adaptive villous branching and capillarisation occur in hypoxic placental environments, including term preeclampsia [43], high altitude [44], smoking [45], and anaemia [46]. Branching angiogenesis is associated with increased vascular endothelial growth factor (VEGF) expression [47], previously shown to be associated with placental malaria parasitaemia and maternal hypertension [41]. Increased villous angiogenesis may explain the previously reported high placental to foetal weight ratio among primigravidae with placental malaria parasitaemia [48].

Primigravidae with early pregnancy malaria parasitaemia had an increased risk of subsequent IUGR, compared to multigravidae with no early pregnancy malaria parasitaemia. Primigravidae are known to have increased severity of malaria infection and worse pregnancy outcomes than multigravidae in malaria endemic areas [24,25]. The findings support limited, but growing, evidence that early pregnancy malaria parasitaemia adversely affects birth weight [49-52] and foetal growth [26]. The adverse effect of early pregnancy malaria parasitaemia on foetal growth in primigravidae appeared to occur despite a blood flow profile consistent with moderate alterations in placentation and increased angiogenesis. This suggests that the effects of early pregnancy malaria parasitaemia on foetal growth among primigravidae occur at the time of infection and may be mediated via mechanistic pathways other than changes in blood flow, such as inflammatory cells and cytokines [53], anaemia [54], or acute changes in blood flow during active parasitaemia [9]. In the current study, a foetus frequently demonstrated signs of IUGR at one or more study time points, yet did not at other time points, suggestive of differential rates of foetal growth during gestation. Further, there was no association between early pregnancy malaria and low birth weight (data not shown), possibly due to the frequent identification and treatment of sub-clinical malaria parasitaemia in the study cohort.

## Conclusion

The prevalence of malaria parasitaemia peaks in early pregnancy [3] and malaria parasitaemia prevention and control measures are infrequently initiated [1] during this critical period in placental development. The effect of early pregnancy malaria parasitaemia on subsequent uterine artery resistance was modified by maternal nutritional status, with increased resistance among undernourished women with early pregnancy malaria and decreased resistance among normally nourished women with early pregnancy malaria. Among primigravidae, early pregnancy malaria parasitaemia decreased umbilical artery resistance in the late third trimester, due to changes in angiogenesis. Thus, early malaria parasitaemia infection may dysregulate placentation and angiogenesis, leading to lasting changes in both uterine and umbilical artery blood flows. Among primigravidae, early pregnancy malaria parasitaemia was associated with nearly four times the risk of subsequent intrauterine growth restriction compared to multigravidae with no early malaria infection. These findings support the initiation of malaria parasitaemia prevention and control efforts earlier in pregnancy.

## Abbreviations

ITN: Insecticide-treated nets; IPTp: Intermittent preventive treatment in pregnancy; IUGR: Intrauterine growth restriction; SES: Socioeconomic status; IQR: Interquartile range; qPCR: Quantitative real-time polymerase chain reaction; RI: Resistance index; MUAC: Mid upper arm circumference; LME: Linear mixed effect; RR: Risk ratio; GEE: Generalized estimating equation; -2LL: -2 log-likelihood.

## Competing interests

The authors declare there are no competing interests.

## Authors’ contributions

JBG developed the objectives, analysed and interpreted the data, and drafted the manuscript. JBG was supported by the Infectious Disease Epidemiology Training Program grant (5-T32-AI070114-04). VL conducted all Doppler ultrasound measurements and interpreted the Doppler data. SHL designed and implemented the study while at the Department of Epidemiology, University of North Carolina – Chapel Hill and contributed to manuscript revision while at GlaxoSmithKline. AHH assisted with statistical analyses and helped prepare the Methods section of the manuscript. AKT supervised field activities, oversaw quality control at the University of Kinshasa and interpreted the data. JMT, SJR and SRM made substantial contributions to the interpretation of results and manuscript revision. All authors read and approved the final manuscript.

## References

[B1] A Strategic Framework for Malaria Prevention and Control During Pregnancy in the African Region 2004 WHO Regional Office for Africa, Brazzaville URL: http://www.who.int/malaria/publications/atoz/afr_mal_04_01/en/index.html

[B2] O'Dowd MJ O'Dowd TM Quickening—a re-evaluation Br J Obstet Gynaecol 1985 92 1037 1039 10.1111/j.1471-0528.1985.tb02999.x 3902075

[B3] Brabin BJ An analysis of malaria in pregnancy in Africa Bull World Health Organ 1983 61 1005 1016 6370484 PMC2536236

[B4] Prefumo F Sebire NJ Thilaganathan B Decreased endovascular trophoblast invasion in first trimester pregnancies with high-resistance uterine artery Doppler indices Hum Reprod 2004 19 206 209 10.1093/humrep/deh037 14688183

[B5] Sebire NJ Sepulveda W Correlation of placental pathology with prenatal ultrasound findings J Clin Pathol 2008 61 1276 1284 10.1136/jcp.2008.055251 18682416

[B6] Trudinger BJ Giles WB Cook CM Uteroplacental blood flow velocity-time waveforms in normal and complicated pregnancy Br J Obstet Gynaecol 1985 92 39 45 10.1111/j.1471-0528.1985.tb01046.x 3966989

[B7] Giles WB Trudinger BJ Baird PJ Fetal umbilical artery flow velocity waveforms and placental resistance: pathological correlation Br J Obstet Gynaecol 1985 92 31 38 10.1111/j.1471-0528.1985.tb01045.x 3966988

[B8] Trudinger BJ Giles WB Cook CM Bombardieri J Collins LEE Fetal umbilical artery flow velocity waveforms and placental resistance: clinical significance Br J Obstet Gynaecol 1985 92 23 30 4038455 10.1111/j.1471-0528.1985.tb01044.x

[B9] Dorman EK Shulman CE Kingdom J Bulmer JN Mwendwa J Peshu N Marsh K Impaired uteroplacental blood flow in pregnancies complicated by falciparum malaria Ultrasound Obstet Gynecol 2002 19 165 170 10.1046/j.0960-7692.2001.00545.x 11876809

[B10] Landis SH A longitudinal ultrasound study of fetal growth and intrauterine growth restriction in Kinshasa 2007 Democratic Republic of Congo. Dissertation. University of North Carolina, Epidemiology

[B11] Landis SH Lokomba V Ananth CV Atibu J Ryder RW Hartmann KE Thorp JM Tshefu A Meshnick SR Impact of maternal malaria and under-nutrition on intrauterine growth restriction: a prospective ultrasound study in Democratic Republic of Congo Epidemiol Infect 2009 137 294 304 10.1017/S0950268808000915 18588723

[B12] Taylor SM Juliano JJ Trottman PA Griffin JB Landis SH Kitsa P Tshefu AK Meshnick SR High-throughput pooling and real-time PCR-based strategy for malaria detection J Clin Microbiol 2010 48 512 10.1128/JCM.01800-09 19940051 PMC2815636

[B13] Ohrt C Obare P Nanakorn A Adhiambo C Awuondo K O'Meara W Remich S Martin K Cook E Chretien J-P Lucas C Osoga J McEvoy P Owaga ML Odera JS Ogutu B Establishing a malaria diagnostics centre of excellence in Kisumu Kenya. Malar J 2007 6 79 10.1186/1475-2875-6-79 PMC1933544 17565676

[B14] Maulik D Maulik DZ, Zalud I Spectral Doppler Sonography: waveform analysis and hemodynamic interpretation Doppler Ultrasound in Obstetrics and Gynecology. 2nd rev. and enlarged ed. edition 2005 Springer-Verlag, Berlin Heidelberg 35 56

[B15] Hadlock FP Harrist RB Martinez-Poyer J In utero analysis of fetal growth: a sonographic weight standard Radiology 1991 181 129 133 1887021 10.1148/radiology.181.1.1887021

[B16] Johnsen SL Rasmussen S Wilsgaard T Sollien R Kiserud T Longitudinal reference ranges for estimated fetal weight Acta Obstet Gynecol Scand 2006 85 286 297 10.1080/00016340600569133 16553175

[B17] Landis SH Ananth CV Lokomba V Hartmann KE Thorp JM Jr Horton A Atibu J Ryder RW Tshefu A Meshnick SR Ultrasound derived fetal size nomogram for a sub Saharan African population: a longitudinal study Ultrasound Obstet Gynecol 2009 34 379 386 10.1002/uog.6357 19402076

[B18] Hadlock FP Deter RL Harrist RB Park SK Estimating fetal age: computer-assisted analysis of multiple fetal growth parameters Radiology 1984 152 497 501 6739822 10.1148/radiology.152.2.6739822

[B19] Mongelli M Wilcox M Gardosi J Estimating the date of confinement: ultrasonographic biometry versus certain menstrual dates Am J Obstet Gynecol 1996 174 278 281 10.1016/S0002-9378(96)70408-8 8572021

[B20] Krasovec K Anderson MA Maternal nutrition and pregnancy outcomes: anthropometric assessment 1991 Washington, DC: Pan American Health Organization, Pan American Sanitary Bureau, Regional Office of the World Health Organization

[B21] Laird NM Ware JH Random-effects models for longitudinal data Biometrics 1982 38 963 974 10.2307/2529876 7168798

[B22] Liang KY Zeger SL Longitudinal data analysis using generalized linear models Biometrika 1986 73 13 10.1093/biomet/73.1.13

[B23] Fitzmaurice GM Longitudinal data analysis 2009 Chapman & Hall/CRC, Boca Raton, FL

[B24] Desai M ter Kuile FO Nosten F McGready R Asamoa K Brabin B Newman RD Epidemiology and burden of malaria in pregnancy Lancet Infect Dis 2007 7 93 104 10.1016/S1473-3099(07)70021-X 17251080

[B25] Rogerson SJ Hviid L Duffy PE Leke RF Taylor DW Malaria in pregnancy: pathogenesis and immunity Lancet Infect Dis 2007 7 105 117 10.1016/S1473-3099(07)70022-1 17251081

[B26] Rijken MJ Papageorghiou AT Thiptharakun S Kiricharoen S Dwell SLM Wiladphaingern J Pimanpanarak M Kennedy SH Nosten F McGready R Ultrasound evidence of early fetal growth restriction after maternal malaria infection PLoS One 2012 7 e31411 10.1371/journal.pone.0031411 22347473 PMC3276538

[B27] Arbeille P Carles G Bousquet F Body G Lansac J Fetal cerebral and umbilical artery blood flow changes during pregnancy complicated by malaria J Ultrasound Med 1998 17 223 229 9544605 10.7863/jum.1998.17.4.223

[B28] Genbacev O Zhou Y Ludlow JW Fisher SJ Regulation of human placental development by oxygen tension Science 1997 277 1669 10.1126/science.277.5332.1669 9287221

[B29] Ordi J Menendez C Ismail MR Ventura PJ Palacín A Kahigwa E Ferrer B Cardesa A Alonso PL Placental malaria is associated with cell-mediated inflammatory responses with selective absence of natural killer cells J Infect Dis 2001 183 1100 10.1086/319295 11237836

[B30] Yui J Garcia-Lloret M Wegmann TG Guilbert LJ Cytotoxicity of tumour necrosis factor-alpha and gamma-interferon against primary human placental trophoblasts Placenta 1994 15 819 835 10.1016/S0143-4004(05)80184-5 7886023

[B31] Renaud SJ Postovit LM Macdonald-Goodfellow SK McDonald GT Caldwell JD Graham CH Activated macrophages inhibit human cytotrophoblast invasiveness in vitro Biol Reprod 2005 73 237 10.1095/biolreprod.104.038000 15800179

[B32] Williams PJ Bulmer JN Innes BA Broughton Pipkin F Possible roles for folic acid in the regulation of trophoblast invasion and placental development in normal early human pregnancy Biol Reprod 2011 84 1148 1153 10.1095/biolreprod.110.088351 21349824

[B33] Brabin BJ Alexander Fletcher K Brown N Do disturbances within the folate pathway contribute to low birth weight in malaria? Trends Parasitol 2003 19 39 43 10.1016/S1471-4922(02)00004-1 12488225

[B34] Conroy AL McDonald CR Silver KL Liles WC Kain KC Complement activation: a critical mediator of adverse fetal outcomes in placental malaria? Trends Parasitol 2011 27 294 299 10.1016/j.pt.2011.02.005 21493146

[B35] Girardi G Bulla R Salmon JE Tedesco F The complement system in the pathophysiology of pregnancy Mol Immunol 2006 43 68 77 10.1016/j.molimm.2005.06.017 16023727

[B36] Burton GJ Jauniaux E Charnock-Jones DS The influence of the intrauterine environment on human placental development Int J Dev Biol 2010 54 303 312 10.1387/ijdb.082764gb 19757391

[B37] Belkacemi L Nelson DM Desai M Ross MG Maternal undernutrition influences placental-fetal development Biol Reprod 2010 83 325 331 10.1095/biolreprod.110.084517 20445129

[B38] Roberts CT IFPA Award in Placentology Lecture: Complicated interactions between genes and the environment in placentation, pregnancy outcome and long term health Placenta 2010 31 S47 S53 20096927 10.1016/j.placenta.2010.01.001

[B39] Myatt L Placental adaptive responses and fetal programming J Physiol 2006 572 25 30 16469781 10.1113/jphysiol.2006.104968 PMC1779654

[B40] Landis SH A longitudinal ultrasound study of fetal growth and intrauterine growth restriction in Kinshasa, Democratic Republic of Congo 2008 Chapel Hill: The University of North Carolina

[B41] Muehlenbachs A Mutabingwa TK Edmonds S Fried M Duffy PE Hypertension and maternal-fetal conflict during placental malaria PLoS Med 2006 3 e446 10.1371/journal.pmed.0030446 17105340 PMC1635741

[B42] Abramowicz JS Sheiner E Ultrasound of the placenta: a systematic approach. Part II: Functional assessment (Doppler) Placenta 2008 29 921 929 10.1016/j.placenta.2008.08.010 18799213

[B43] Kingdom JCP Kaufmann P Oxygen and placental villous development: Origins of fetal hypoxia Placenta 1997 18 613 621 10.1016/S0143-4004(97)90000-X 9364596

[B44] Jackson MR Mayhew TM Haas JD Morphometric studies on villi in human term placentae and the effects of altitude, ethnic grouping and sex of newborn Placenta 1987 8 487 495 10.1016/0143-4004(87)90077-4 3422920

[B45] Pfarrer C Macara L Leiser R Kingdom J Adaptive angiogenesis in placentas of heavy smokers Lancet 1999 354 303 10.1016/S0140-6736(99)01676-1 10440311

[B46] Kadyrov N Kosanke G Increased fetoplacental angiogenesis during first trimester in anaemic women Lancet 1998 352 1747 1749 10.1016/S0140-6736(98)02069-8 9848351

[B47] Cao Y Linden P Shima D Browne F Folkman J In vivo angiogenic activity and hypoxia induction of heterodimers of placenta growth factor/vascular endothelial growth factor J Clin Invest 1996 98 2507 10.1172/JCI119069 8958213 PMC507708

[B48] Brabin BJ Romagosa C Abdelgalil S Menendez C Verhoeff FH McGready R Fletcher KA Owens S D'Alessandro U Nosten F The sick placenta-the role of malaria Placenta 2004 25 359 378 10.1016/j.placenta.2003.10.019 15081631

[B49] Cottrell G Mary JY Barro D Cot M The importance of the period of malarial infection during pregnancy on birth weight in tropical Africa AmJTrop Med Hyg 2007 76 849 17488903

[B50] Huynh BT FN Gbaguidi G Dechavanne S Borgella S Guezo-Mevo B Massougbodji A Ndam N Deloron P Cot M Influence of the timing of malaria infection during pregnancy on birth weight and on maternal anaemia in Benin AmJTrop Med Hyg 2011 85 214 220 10.4269/ajtmh.2011.11-0103 PMC3144815 21813837

[B51] Kalilani L Mofolo I Chaponda M Rogerson SJ Meshnick SR The effect of timing and frequency of Plasmodium falciparum infection during pregnancy on the risk of low birth weight and maternal anemia Trans R Soc Trop Med Hyg 2010 104 416 422 10.1016/j.trstmh.2010.01.013 20207387 PMC4844554

[B52] Taha TET Gray RH Mohamedani AA Malaria and low birth weight in central Sudan Am J Epidemiol 1993 138 318 8356970 10.1093/oxfordjournals.aje.a116861

[B53] Fried M Muga RO Misore AO Duffy PE Malaria elicits type 1 cytokines in the human placenta: IFN-{gamma} and TNF-{alpha} associated with pregnancy outcomes J Immunol 1998 160 2523 9498798

[B54] Menendez C Ordi J Ismail MR Ventura PJ Aponte JJ Kahigwa E Font F Alonso PL The impact of placental malaria on gestational age and birth weight J Infect Dis 2000 181 1740 1745 10.1086/315449 10823776

